# Identification and expression analysis of a microRNA cluster derived from pre-ribosomal RNA in *Papaver somniferum* L. and *Papaver bracteatum* L.

**DOI:** 10.1371/journal.pone.0199673

**Published:** 2018-08-01

**Authors:** Farshad Davoodi Mastakani, Gabriel Pagheh, Sajad Rashidi Monfared, Masoud Shams-Bakhsh

**Affiliations:** 1 Department of Agricultural Biotechnology, Faculty of Agriculture, Tarbiat Modares University, Tehran, Iran; 2 Department of Plant Pathology, Faculty of Agriculture, Tarbiat Modares University, Tehran, Iran; Dokuz Eylul Universitesi, TURKEY

## Abstract

Opium poppy (*Papaver somniferum* L.) is one of the ancient medical crops, which produces several important alkaloids such as morphine, noscapine, sanguinarine and codeine. MicroRNAs are endogenous non-coding RNAs that play important regulatory roles in plant diverse biological processes. Many plant miRNAs are encoded as single transcriptional units, in contrast to animal miRNAs, which are often clustered. Herein, using computational approaches, a total of 22 miRNA precursors were identified, which five of them were located as a clustered in pre-ribosomal RNA. Afterward, the transcript level of the precursor and the mature of clustered miRNAs in two species of the Papaveraceae family, i.e. *P*. *somniferum* L. and *P*. *bracteatum* L, were quantified by RT-PCR. With respect to obtained results, these clustered miRNAs were expressed differentially in different tissues of these species. Moreover, using target prediction and Gene Ontology (GO)-based on functional classification indicated that these miRNAs might play crucial roles in various biological processes as well as metabolic pathways. In this study, we discovered the clustered miRNA derived from pre-rRNA, which may shed some light on the importance of miRNAs in the plant kingdom.

## Introduction

*Papaver somniferum* L., a member of Papaveraceae family, is one of the most agronomically and economically important medical crops with powerful pharmacological features [1]. The opium poppy produces numerous secondary metabolites such as benzylisoquinoline alkaloids (BIAs) including noscapine, morphine, codeine, papaverine and sanguinarine [2]. Recently, microRNAs (miRNAs) have emerged as tiny RNA mediators with a plethora of key biological functions in plants; such as regulation of gene expression at the post-transcriptional level that either transcript cleavage or translation inhibition of targeting mRNAs [3, 4]. In plants, miRNAs play pivotal regulatory roles in many biological processes including response to environmental stresses such as drought [5], salinity [6], chilling [7], mechanical damage [8] and developmental processes [9] like flower and root morphogenesis [10, 11].

Plant miRNAs derived from an intragenic region of the genome that is transcribed as pri-miRNAs. These miRNAs may be clustered in a transcript unit, which converts to precursor miRNA (pre-miRNA), when is affected by a ribonuclease enzyme Dicer like1 enzyme (DCL1). The pri-miRNAs contain self-complementary sequences and upon folding, form an internal stem-loop structure with 3' and 5' tails. Subsequently, pri-miRNAs cleave and form miRNA:miRNA* duplex [12]. This duplex transported to the cytoplasm by HASTY protein. One of the strands is to be degraded which called miRNA*. Whereas, mature miRNA strand is loaded into the RNA-induced silencing complex (RISC); single-stranded miRNAs guide the RISC complex to connect their target genes by a complementary approch or a near complementary [4]. The mentioned steps describe the canonical pathway of miRNA biogenesis. However, recent studies show non-canonical pathways of miRNA production or any ways to generate miRNA-like, for example, Drosha-independent pathways including Mirtron, snoRNA, shRNA and tRNA, pre-siRNA, tRNase Z-dependent pathway and Dicer-independent pathways including AGO, tRNaseZ- dependent pathway [13].

Pre-rRNA in eukaryote contains 18S, 5.8S, 28S rRNAs and Internal Transcribed Spacer 1 (ITS1), Internal Transcribed Spacer 2 (ITS2). Pre-rRNA processing always possesses important stages including synthesis of precursor rRNA, cleavage of the pre-rRNAs, modification, assembly, and transportation to pre-ribosomes, through the nucleolus and nucleus and quality controls [14]. According to the study conducted by Son et al [15], mmu-miR-712 (*Mus musculus* miR-712) is derived from an unexpected source, pre-ribosomal RNA by exoribonucleases that involved in Pre-rRNA maturation. The nucleotide sequence search revealed that the sequence of pre-miR-712 is located in the ITS2 region of murine pre-ribosomal RNA. Many of known animal miRNAs appear in clusters on single transcript units. Clustered miRNAs can be transcribed into single units either independently or together. In animal species, many miRNAs as a cluster showed similar conserved pattern [16–19], however, few clustered miRNAs are identified in plants [20, 21]. The genome location and biogenesis pathway of clustered miRNAs have not yet known.

Investigation of miRNAs in opium poppy has a relatively short history [22, 23]. The EST sequences can be employed to identify miRNAs and their targets in the plant kingdom. EST sequences have been analyzed for identification and prediction of miRNAs in *Brassica napus* L. [24], *Glycine max* L. [7], *P*. *somniferum* L. [22], *Helianthus annuus* L. [25]. Recently, computational prediction and deep sequencing technology, are the main approaches in order to detection of given miRNA [26]. Computational methods provide a useful tool for mining conserved miRNAs in diverse species, which leaning on the available sequences in the public databases [27]. In this study, we exploited *P*. *somniferum* L. and *P*. *bracteatum* L. transcriptome data and known plant miRNAs so as to identify five clustered miRNAs and 16 miRNAs from different miRNA families using *in silico* approaches. Then, expression profiles of the precursor and mature clustered miRNAs were analyzed.

## Materials and methods

### Data source

In order to identify potential miRNAs, 16438 known plant miRNAs from PNRD database (http://structuralbiology.cau.edu.cn/PNRD/index.php) and several RNA-seq projects for *P*. *somniferum* L. (With SRA accession nos.: SRX118163, SRX117971, SRX013002, SRX012997, SRX012994, SRX012995, SRX012996, SRX012998, SRX012999, SRX013000, SRX013001) and *P*. *bracteatum* L. (With SRA accession nos.: SRX039638, SRX096061) were downloaded from the following database: http://www.ncbi.nlm.nih.gov/sra/.

### Bioinformatics analysis of *P*. *somniferum* L. and *P*. *bracteatum* L. miRNAs

Searching for potential miRNAs was performed in *P*. *somniferum* L. and *P*. *bracteatum* L. by a published method with some modifications as indicated below [28–33]. The sequences of the mature and pre-miRNAs were used as a query sequences for offline BLASTn against the RNA sequences; for mature miRNAs the following parameters such as: e-value cut-off 10, word match size 7 nt, allowed only 0–2 mismatches and for pre-miRNAs the parameters highest percentage match 90 and word match size 28 nt were applied. In addition, mature miRNA sequences should not be less than 17 nt and should be a maximum of 25 nt. The precursor sequences of 300 nt were extracted (150 bp upstream and 150 downstream to the BLASTn hits) and used for hairpin structure prediction. Consequently, we used 300 nt for pre-miRNA [20] and hairpin structure prediction using Mirval web tools (http://mimirna.centenary.org.au/mireval) and Mfold 3.2 software [34] according to the mature miRNA region on the BLAST hits. Then, based on following characters, mature and precursor miRNAs were predicted. Between 60 to 400 nt was used for the length of plant pre-miRNAs [35].

The parameters used for selection of conserved miRNA precursors were as following: i) folding miRNA precursor sequences into a suitable secondary stem-loop structure; ii) no break or loop and lower than six nucleotide mismatches between mature miRNA and its opposite miRNA* sequence; iii) one arm of a precursor stem part includes mature miRNA and its opposite arm strands miRNA* sequence; iv) higher negative Minimal Folding free Energy Index (MFEI) values of candidate precursors miRNA than other species of RNAs; v) minimal free energy allowed for miRNA precursor (−15 kcal.mol^-1^); vi) there is 3 nt asymmetric bulge within the miRNA and miRNA* duplex and the bulge should not be more than 2 nucleotides in size. Also, minimal base pairs of miRNA and miRNA* was considered 16 nt in size [33, 36] and vii) G + C content between 24% and 71%. After that, the precursor candidate sequences were analyzed by BLASTx at default parameter against non-redundant protein sequences (nr) dataset to check potential protein-coding sequence. The confirmed final candidate miRNAs are mapped onto the non-redundant (nr) DNA sequences dataset using the BLASTn tool.

### Target gene identification and functional analysis

The target genes of the identified miRNAs were predicted on the web-based psRNATarget Server (http://plantgrn.noble.org/psRNATarget/). Both Gene Ontology (GO) and Kyoto Encyclopedia of Genes and Genomes (KEGG) analysis were employed to further study the biological processes and the corresponding metabolic pathways regulated by potential miRNAs, respectively. GO analysis was performed using combined agriGO [37] and TAIR website [38]. In addition, putative miRNA targets were used as query sequences against the KEGG database (http://www.genome.jp/kegg) using default settings for pathway analysis [39]. Co-expression networks of putative target genes were also analyzed by ATTED-II (http://atted.jp/) with default parameters.

### Validation of miRNA expression by quantitative RT-PCR

#### Experimental precursor miRNA detection and expression level measurements

Total RNA of *P*. *somniferum* L. and *P*. *bracteatum* L. was extracted using an RNeasy Plant Mini Kit-QIAGEN (Lot No: 142338933) according to manufacturer’s instructions. Then, the quantity and the quality of the extracted RNA were determined using NanoDrop spectrophotometer (BioTek, EPOCH, serial 121004C, USA), and confirmed via agarose gel electrophoresis. RNase-free DNase I (Fermentase Cat. No: EN0521) was used to eliminate genomic DNA content. Afterward, M-MLV Reverse Transcriptase (Solis BioDyne: C101031) was used to synthesize the first-strand cDNA with Random Hexamer and oligo d(T) primers. For cDNA synthesis, a 14 μl reaction mixture containing 5 μl (1 μg) total RNA, 1 μl Random Hexamer primer (10 pmol.μl^-1^), and 8 μl free nuclease water was heated to 75°C (10 min) for denaturing of RNAs hairpin structures, immediately, chill the tube on ice for 5 min. Then a reaction premix containing 2 μl dNTP (10 μm), 2 μl RT reaction buffer (5X) and 1 μl M-MLV Reverse Transcriptase (200U.μl^-1^) were added. Then, we applied 16°C for 15 min followed by 25°C for 30 min. Finally, we added 1 μl oligo dT primer (10 pmol.μl^-1^) and reactions were incubated at 42°C for 60 min and heated to 75°C for 10 min to inactivate the enzyme. ([40], with some modifications). The expected length of miRNA fragments is shown (S3 Fig). In order to amplification of given miRNAs, we used following program: 5 pmol.μl^-1^ forward primer, 5 pmol.μl^-1^ reverse primer, 10 mmol.L^-1^ dNTPs, 100 ng.μl^-1^ cDNA, 5 U. μl^-1^ Taq DNA Polymerase (CinaGen CO., Cat. No. TA7505C, Iran), 10 X Tag Buffer and 10 X MgCl2. The total volume for each reaction was reached to 10 μl. The following thermal cycler was conducted this end: 4 min at 94°C, 30 s at 94°C, 30 s at 54–60°C, 10 s at 72°C for 35 cycles; followed by 10 min 72°C.

The expression levels of precursor clustered miRNAs were measured in stem, root, leaf and capsule tissues of both *P*. *somniferum* L. and *P*. *bracteatum* L. The qRT-PCR was conducted with miRNAs specific primers designed based on the precursor region of each miRNA to ensure specific amplification (Table 1). The qRT-PCR was performed using a Bio-Rad system with the fluorescent dye SYBR Green® 2X Master Mix (Ampliqon, cat No.: A325402, Denmark) in accordance with the manufacture’s recommendation. The following program was used to carry out qRT-PCR: 0.5 μl (5 pmol.μl^-1^) of each primer, 0.5 μl of temple cDNA, 5 μl SYBR®Green Master Mix 2X in a total volume of 10 μl. The qRT-PCR was conducted at 95°C for 15 min, followed by 40 cycles at 95°C (30 s), 58°C (30 s) and 72°C (20 s). Furthermore, the melting curves were adjusted as 60–95°C, 5°C for 5 s. For quantifying transcription levels, the reference gene 5.8S rRNA (GenBank Accession No.: AY328297.1) was used as an internal control. Three technical replicates were performed per sample. The expression levels were calculated using Bio-Rad CFX Manager software, and cycle thresholds (C(t)s) were analyzed using the 2^-ΔΔCt^ method [41].

**Table 1:** The list of primers used for qRT-PCR and Semi RT-PCR for analysis of transcript level of precursor and mature miRNAs in *P*. *somniferum* L. and *P*. *bracteatum* L.

Primer Name	Use	Primer Sequence
MIR1310-F1	Precursor and mature detection	5'-aggcatcgggggcgcaac-3'
MIR1310-R1	Precursor detection	5'-gcggctcattggagcagcc-3'
MIR2911-F1	Precursor detection	5'-cccagtcccgaacccgtc-3'
MIR2911-R1	Precursor detection	5'-tcccaatccgtccccc-3'
MIR2910-F1	Precursor detection	5'-tagttggtggacgatt-3'
MIR2910-R1	Precursor detection	5'-tagcaggctgaggtctc-3'
MIR2916-F1	Precursor and mature detection	5'-tgggggctcgaagacgatcag-3'
MIR2916-R1	Precursor detection	5'-ggcggagtcctaaaagcaacatc-3'
MIR2914-F1	Precursor detection	5'-ttctgccctatcaactttcg-3'
MIR2914-R1	Precursor detection	5'-ctccgtcacccgtcac-3'
5.8S rRNA_F1	Control	5'-aacgactctcggcaacggatatc-3'
5.8S rRNA_R1	Control	5'-aactcgatggttcacgggattc-3'
MIR2914-F2	Mature detection	5'- gtggtgacgggtgacggag-3'
MIR2910-F2	Mature detection	5'-ttggtggagcgatttgtc-3'
MIR2911-F2	Mature detection	5'-cgggggacggctggga-3'
Universal.-R	Mature detection	5'-aagcagtggtatcaacgcagagt-3'
Oligo dTVN	Mature detection	5'-ctaatacgactcactatagggcaagcagtggtatcaacgcagagt(t)18VN-3'

#### Experimental mature miRNA detection and expression pattern assay in different tissues

We used poly(T) adaptor RT-PCR methods for detection and quantitation of mature miRNA expression. Initially, poly(A) tail was added to the 3′ end of miRNAs using poly(A) polymerase (New England BioLabs, Cat. No. M0314S) based on manufacture’s recommendation. Meanwhile, the first strand cDNA was synthesized using 300 ng of given RNA with oligo dT adaptor. The expression of mature miRNAs was measured using RT-PCR with four replications. To this end, RT-PCR was carried out by Bio-Rad system and using the universal primer as reverse and specific primer for mature miRNAs as forward primer (Table 1). Each reaction was performed in a final volume of 10 μl containing 5 μl of PCR Master Mix 2x (Amplicon, Denmark, Cat.No. 180306), 1 μl of cDNA (15 ng), 0.5 μl of miRNA-specific forward primer (10 pmol.μl^-1^) and 0.5 μl universal reverse primer (10 pmol.μl^-1^) and 3 μl nuclease-free water. The PCR was programmed as follows: initial denaturation at 95°C for 5 min, followed by 36 cycles of denaturation at 94°C for 3 min, annealing at 58°C for 30 s, extension at 72°C for 12 s, and a final elongation at 72°C for 5 min. PCR reaction mixtures without template were utilized as negative controls and the 5.8S rRNA was used as internal reference in the RT-PCR assay. PCR products were separated by electrophoresis on a 3% agarose gel. Subsequently, the images were captured by Gel Doc (Uvitec, serial 0510688, UK) and band densities were quantified by image densitometry using the ImageJ 1.44p software (National Institutes of Health, USA). Finally, relative expression levels were calculated by Microsoft Excel software (Microsoft Office 2013), and P value was also calculated using the Fisher exact test with SAS software (SAS Institute, Cary, NC, USA).

## Results

### Identification of miRNAs in *P*. *somniferum* L. and *P*. *bracteatum* L

**Fig 1:**
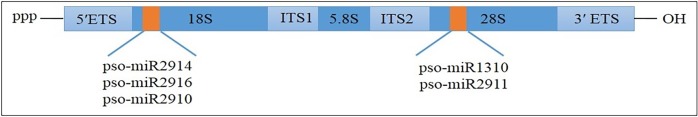
Schematic representations of cluster miRNAs in pre-rRNA of *P*. *somniferum* L. and *P*. *bracteatum* L. ETS: External Transcribed Spacer, ITS: Internal Transcribed Spacer.

**Fig 2:**
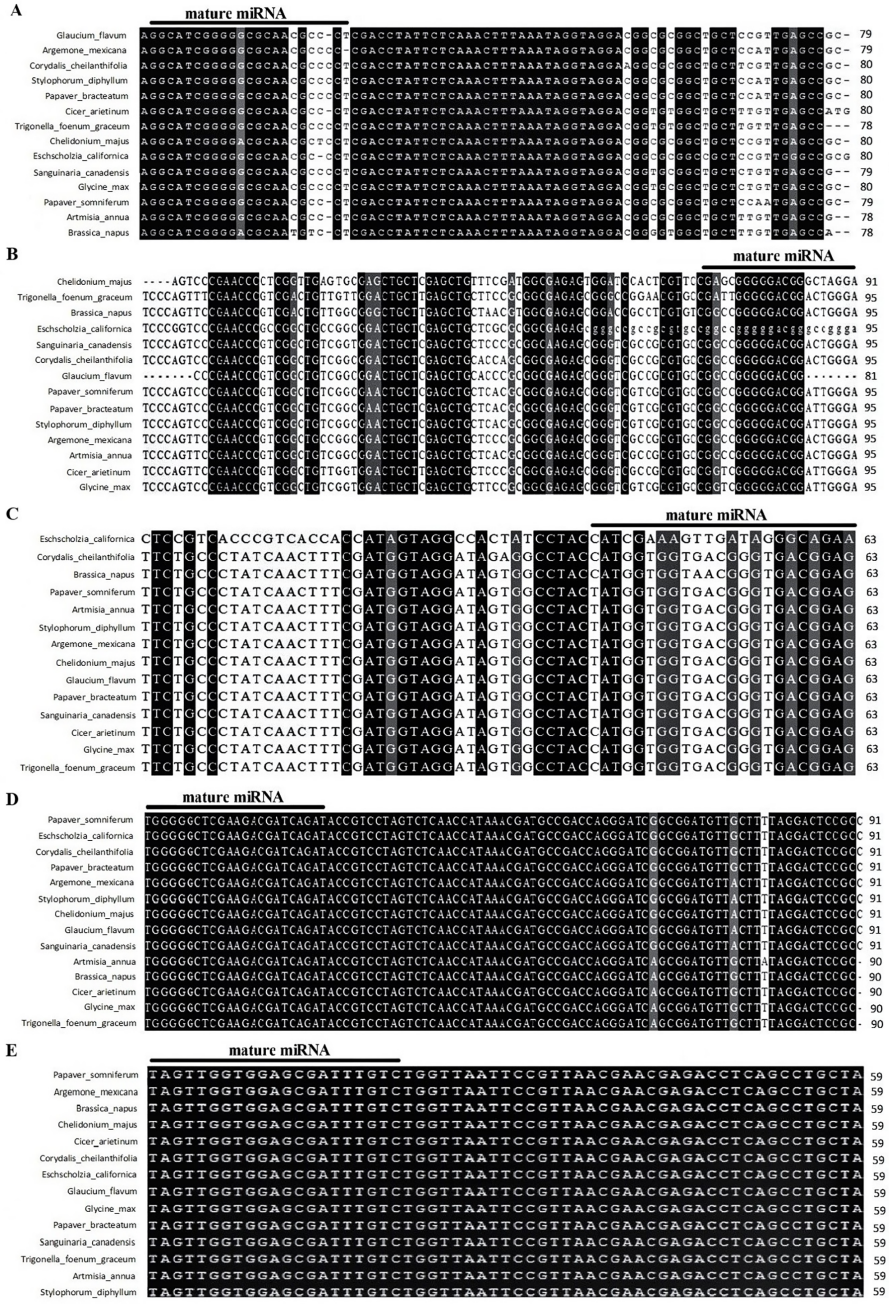
Multiple sequence alignments of clustered miRNA precursors. Sequence alignment of MIR1310 (A), MIR2911 (B), MIR2914 (C), MIR2916 (D) and MIR2910 (E) in C. *aritinum*, *G*. *max*, *P*. *bractatum* L., *P*. *somniferum* L., *A*. *mexicana* L., *A*. *annua* L., *B*. *napus* L., *C*. *majus* L., *C*. *cheilanthifolia*, *E*. *californica* Cham, *G*. *flavum* Crantz., *S*. *canadensis* L., *S*. *diphyllum* Nutt., *T*. *foenum-graceum* L.

Mature miRNAs in plant species are evolutionarily conserved [42], whereas, precursor miRNAs are not conserved and vary from species to species in the plant kingdom. In this study, we applied *in silico* approaches to discover of conserved miRNAs in *P*. *somniferum* L. and *P*. *bracteatum* L. transcriptome. With respect to obtained results, a total of 22 miRNAs were predicted from *P*. *somniferum* L. and *P*. *bracteatum* L. (Table 2). The length of precursor miRNAs ranged from 59 to 164 nt, with an average length of 88 nt (Table 2). Moreover, the precursor miRNAs had G + C content of 27.06% to 72.63% with an average of 48.84%. The MFE and MFEI are important criteria to characterize the stability of the complete or near-complete secondary hairpin structure of the pre-miRNAs [43]. The average MFE value of miRNAs hairpin structures is -32.63 kcal.mol^-1^ with a range of -14.20 kcal.mol^-1^ to -67.70 kcal.mol^-1^. In addition, MFEI for predicted miRNAs ranged from -1.03 to -0.49 with an average of -0.74 (Table 2). For 21 miRNA duplex-like pairs, we predicted, 10 miRNAs anchored in the 5p-arm and 11 miRNA sequences in the 3p-arm. Secondary structures of Papaver miRNAs are shown in S1 Fig. The result of mapping different miRNAs using BLASTn tool showed, interestingly, that the five of miRNAs mapped on well-characterized long polycistronic pre-ribosomal RNAs in different species such as *Coopernookia polygalacea* (KP828778.1), *Panax ginseng (*KM036295.1), *Panax quinquefolius* (KM036297.1), *Goodenia viscida* (KP828797.1), *Coopernookia strophiolata* (KP828785.1). This cluster includes five miRNAs named miR2910, miR2911, miR2914, miR2916 and miR1310. According to the mapping analysis, the exact positions of clustered miRNA on pre-rRNA was characterized as all miRNAs are located in 18S subunit (including miR2914, miR2916, miR2910) and 28S subunit (including miR1310, miR2911) (Fig 1) (S2 Fig). Due to the unavailability of complete genome and pre-rRNA sequences of Papaveraceae family and many other species, NCBI's Sequence Read Archive (SRA) database was searched for finding transcriptomes data sets derived from various RNA-seq projects from *P*. *somniferum* L. and some other plant species such as *Cicer aritinum* L., *Glycin max* L., *Artemisia annua* L., *Brassica napus* L., *P*. *bracteatum* L., *Argemone mexicana* L., *Chelidonium majus* L., *Corydalis cheilanthifolia*, *Eschscholzia californica* Cham., *Glaucium flavum* Crantz., *Sanguinaria canadensis* L., *Stylophorum diphyllum* Nutt., *Trigonella foenum-graecum* L., which are similar to a given query pre-rRNA by BLASTn search tool. Subsequently, the sequence reads were assembled to build up contigs containing pre-rRNA sequence by using the Codon Code Aligner v. 5.0.1. program. After the assembly, sequences of miRNA precursor in pre-rRNA sequences from different species were separated. In order to perform an evolutionary analysis of the clustered miRNAs, we compared the precursor sequences of the miRNAs in studied different plant species utilizing the online Clustal Omega program (http://www.ebi.ac.uk/Tools/msa/clustalomega/). The result showed that the precursors of the miRNAs were highly similar among various plant species (Fig 2), which suggests that clustered miRNAs would play important and crucial roles in the various physiological processes of the species. The mature sequences of miR2910, miR2911, miR2914 and miR2916 were identified from *Populus euphratica* Oliv. [44]. With respect to the large number of genomic scaffolds available from existing whole-genome shotgun (WGS) assembly project of *P*. *euphratica* Oliv, the mapping of assembled pre-rRNA sequence of *P*. *somniferum* L. which contains the clustered miRNAs, into scaffold sequences of *P*. *euphratica* Oliv. was performed by using the BLASTn tool. The result of mapping demonstrated that pre-miRNA of *P*. *somniferum* L. were successfully mapped, and high-level of similarity between sequences were detected. There are no reported precursor miRNAs from *P*. *somniferum* L. and *P*. *breacteatum* L. in the miRNAs database such as plant miRNA database (PMRD) and microRNA database (miRBase). However, recently a total of 327 mature miRNAs have been isolated from *P*. *somniferum* L. using small RNAs sequencing and direct cloning methods [23]. The mature of three out of five clustered miRNAs (miR2910, miR2916 and miR1310) were observed within the above-identified mature miRNAs.

**Table 2:** List of miRNAs identified in two species of *P*. *somniferum* L. and *P*. *bracteatum* L.

miRNA Name	Mature miRNA Sequence	*P*. *somniferum* L.	*P*. *bracteatum* L.	A + U %	G + C %	LP	MFE	MFEI
pso-miR1310	GGCAUCGGGGGCGUAACGCCCCU	✓	✓	40.51	59.49	80	-32.80	-0.68
pso-miR2911	GGCCGGGGGACGGGCUGGGA	✓	✓	27.37	72.63	95	-62.80	-0.91
pso-miR2910	UAGUUGGUGGAGCGAUUUGUC	✓	✓	52.54	47.46	59	-15.51	-0.55
pso-miR2916	UGGGGACUCGAAGACGAUCAUAU	✓	✓	47.83	52.17	98	-27.40	-0.53
pso-miR2914	CAUGGUGGUGACGGGUGACGGAG	✓	✓	47.62	52.38	63	-19.20	-0.58
pso-miR156h	UUGACAGAAGAUAGAGAGCAC	✓		53.26	46.74	92	-44.30	-1.03
pso-miR172b	AGAAUCUUGAUGAUGCUGCAU	✓		55.22	43.28	134	-41.70	-0.71
pso-miRf11320-akr	AAGAUGGAGAAGCAGGGCACGUGC	✓		54.78	45.22	115	-38.10	-0.73
pso-miRf12412-akr	GCUGGGAUUACAGGCGUGAGCCACC	✓		41.18	58.82	85	-28.50	-0.57
pso-miRf12256-akr	CACCAAAGGCCUCUGCCCUUC	✓	✓	42.19	57.81	64	-32.80	-0.88
pso-miR398b	UGUGUUCUCAGGUCGCCCCUG	✓		54.55	45.45	99	-40.90	-0.90
pso-miR1122	UACUCCCUCCGUCCGAAAUUAUUU	✓	✓	64.71	35.29	85	-19.20	-0.64
pso-miR415	AACAGAGCAGAAACAGAA	✓	✓	69.41	30.59	85	-20.60	-0.79
pso-miR414	UCAUCUUCAUCAUCAUCGUCA	✓		64.94	35.06	77	-14.20	-0.49
pso-miR5023	AUUGGUAGUGGAUAAGGGGGC	✓		62.35	37.65	85	-21.30	-0.92
pso-miR1134	ACAACAACAACAAGAAGAA	✓		72.94	27.06	85	-23.70	-0.66
pso-miR6485	UAGGAUGUAGAAGAGCAUAA	✓	✓	57.65	42.35	85	-19.30	-0.53
pso-miRf10082-akr	GGUGCAGGUGCAGGUGCAG	✓	✓	39.81	60.19	103	-52.10	-0.84
pso- miR168	UCGCUUGGUGCAGGUCGGGA	✓		28.57	71.43	119	-67.70	-0.79
pbr-miR408	AUGCACUGCCUCUUCCCUGGC		✓	49.41	50.59	85	-24.80	-0.57
pbr-miRf12309-akr	CACCAAAGGCCUCUGCCCUUC		✓	45.88	54.12	85	-38.40	-0.83
pso-miR170	UGAUUGAGCCGUGCCAAUAUC	✓		62.6	37.4	111	-40.90	-0.98

LP, length of pre-miRNA; MFE, minimal folding-free energy; MFEI, minimal folding-free energy index

### RT-qPCR analysis of clustered precursor miRNAs in *P*. *somniferum* L. and *P*. *bracteatum* L

**Fig 3:**
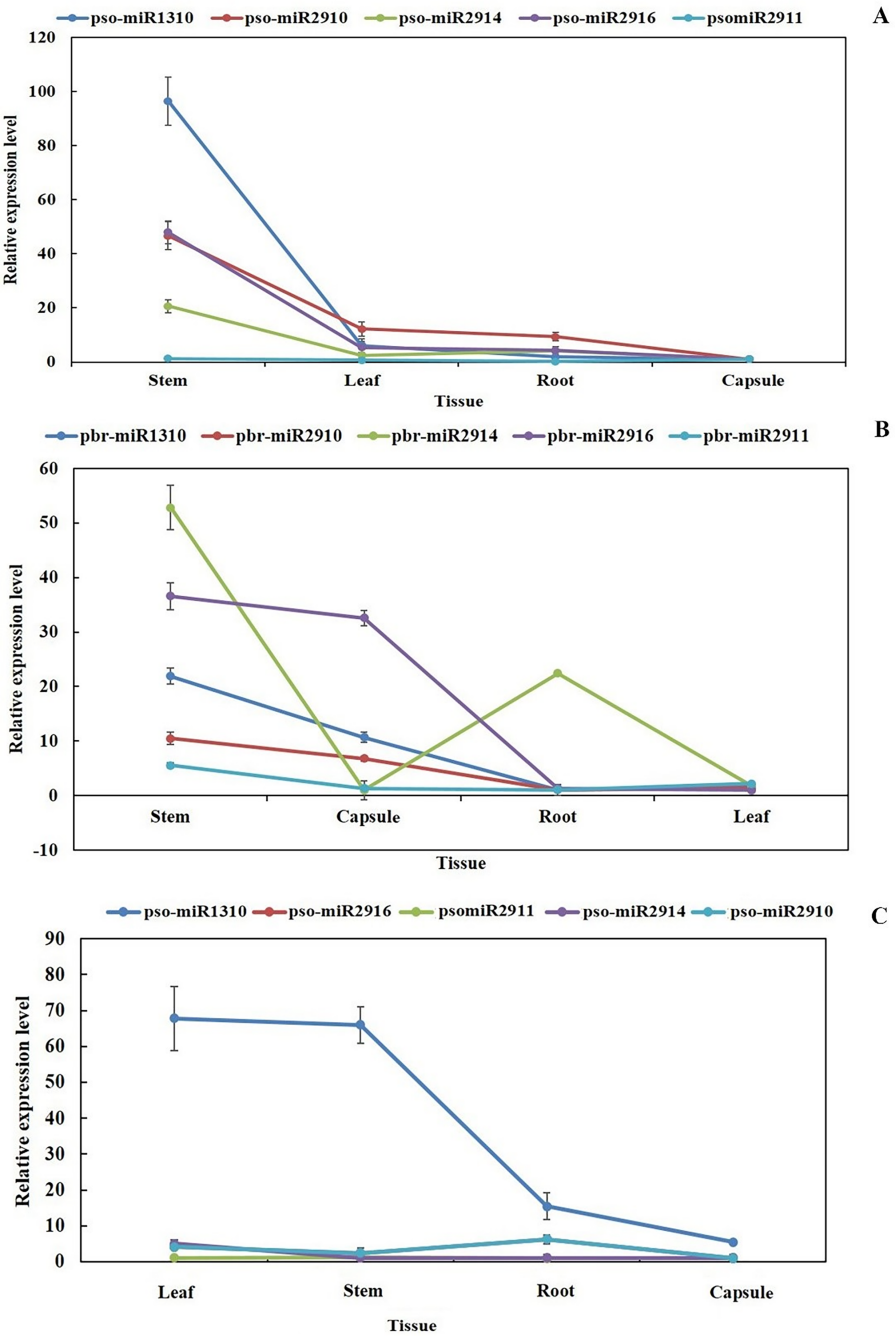
Quantitative RT-PCR assays for the expression of clustered precursor miRNAs are shown. (A) Relative expression patterns in leaf, stem and leaf tissues of *P*. *somniferum* L. in comparison with capsule tissue and (B) Relative expression patterns in stem, capsule and root of *P*. *bracteatum* L. in comparison with leaf tissue (C) Relative expression patterns in leaf, stem, root and capsule of *P*. *somniferum* L. in comparison with those tissues in *P*. *bracteatum* L. 5.8S rRNA was used as the internal control. This analysis verified the differential expression of pre-miRNAs cluster in both of *P*. *somniferum* L. and *P*. *bracteatum* L. pso: *P*. *somniferum* L., pbr: *P*. *bracteatum* L.

In the present study, RT-qPCR was used to study five miRNAs expression patterns in *P*. *somniferum* L. and *P*. *bracteatum* L. tissues using 5.8S rRNA as the internal reference gene. The RT-qPCR analysis showed that all the clustered miRNAs were expressed in root, stem, leaf and capsule tissues of both species. Among them, miR2910, miR2914, miR2916 and miR1310 were mostly expressed in young stem and leaf tissues in both *P*. *somniferum* L. and *P*. *bracteatum* L.; however, we also observed extremely low expression level of miR2911 in all tissues of both species (Fig 3). In this study, 5 unique miRNAs showed differential expression patterns in stem, root, leaf and capsule tissues of *P*. *somniferum* L. and *P*. *bracteatum* L. (Fig 3A and 3B). Furthermore, the highest expression levels of clustered miRNAs were observed in stem tissue of both species. While the lowest expression levels of all miRNAs were observed in capsule of *P*. *somniferum* L. and leaves of *P*. *bracteatum* L. Among studied miRNAs, pso-miR1310 showed the highest expression levels in stem compared to other tissues in *P*. *somniferum* L. Unlikely, *P*. *somniferum* L., pbr-miR2914 had the highest transcription level in stem and root tissues of *P*. *bracteatum* L. (Fig 3C).

Comparison of transcript levels in both species, showed that expression levels of pso-miR1310 in leave, stem and root of *P*. *somniferum* L. were 67.8, 66.02 and 15.44 times more than their expression in same tissues of *P*. *bracteatum* L., respectively. however, the expression level of other miRNAs in all tissues of *P*. *somniferum* L. were almost similar to *P*. *bracteatum* L. (Fig 3C). According to a conducted research in *P*. *somniferum* L., using the high-throughput sRNA-sequencing method, the high expression levels of miR2916, miR2911 and moderate expression level of miR1310 were observed in all mixed tissues of *P*. *somniferum* L. Moreover, the result of miRNA microarrays expression patterns also indicated that several miRNAs showed differential expression among different tissues. Furthermore, the high expression of the miR2916 occurred in root and stem than that was in leaf and capsule tissues, and high expression of miR2911 in leaf [23].

### Semi RT-PCR analysis of clustered mature miRNAs in *P*. *somniferum* L. and *P*. *bracteatum* L

**Fig 4:**
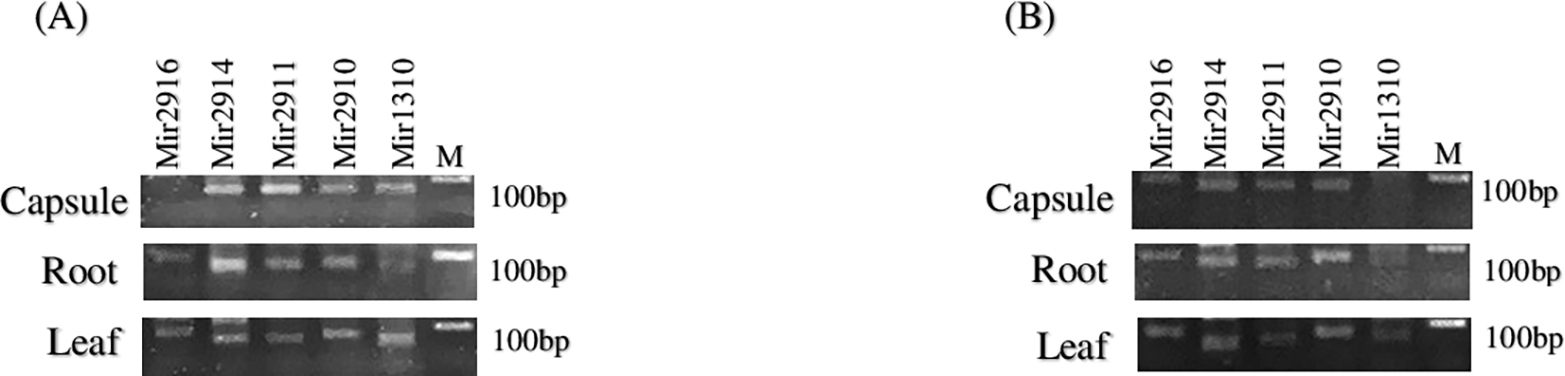
Semi-quantitative RT-PCR showing expression of clustered miRNAs in *P*. *somniferum* L. (A) and *P*. *bracteatum* L. (B). M: 100‐bp DNA ladder (SMOBIO: dm2300).

**Fig 5:**
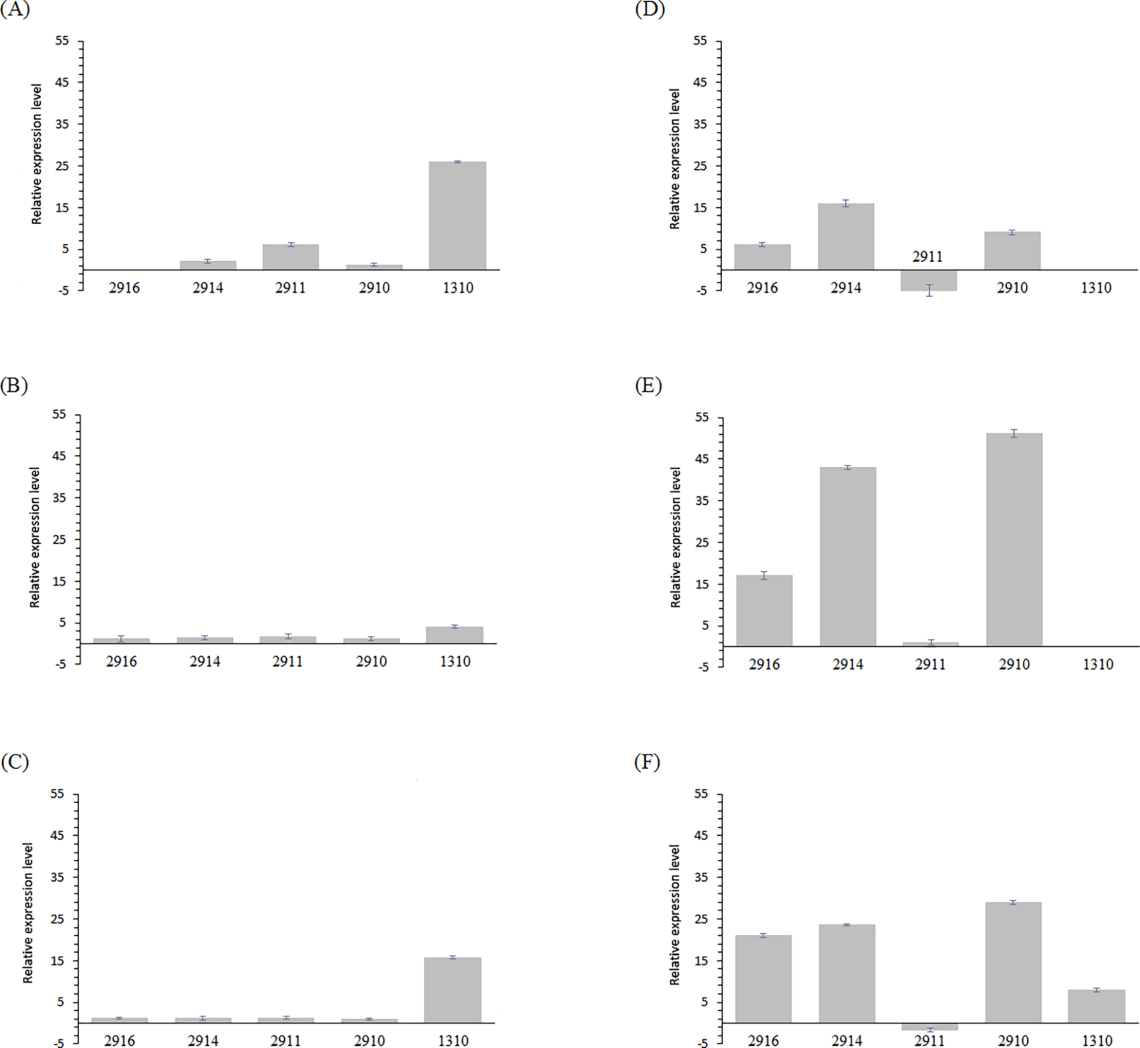
The relative expression level of clustered mature miRNAs evaluated by the semi-quantitative RT-PCR method. The relative expression levels in the capsule (A), root (B) and leaf (C) tissues of *P*. *somniferum* L. in comparison to stem tissue as control, and capsule (D), root (E) and leaf (F) tissues of *P*. *bracteatum* L. in comparison to stem tissue as a control. 5.8S rRNA was used as the reference gene.

To test our hypothesis that the five clustered mature miRNAs are expressed in the both of *P*. *somniferum* L. *and P*. *bracteatum* L. species, we performed mature miRNA profiling on four tissues, including stem, root, leaf and capsule, of these species by semi-quantitative RT-PCR. Afterward, miRNAs expression data was normalized to the expression level of 5.8S rRNA. In this study, RT-PCR results showed differential expression pattern of the mature miRNAs in various tissues. The highest expression level of clustered miRNAs was observed in capsule, root, leaves and stem tissues of *P*. *somniferum* L., respectively. In contrast, root, leaves, capsule and stem tissues of *P*. *bracteatum* L. had the highest expression level, respectively (Figs 4 and 5). In addition, miR2910, miR2911 and miR2914 were expressed in all tissues of *P*. *somniferum* L., whereas, miR2916 were undetectable in capsule tissues of *P*. *somniferum* L. In another hand, only miR2911 was expressed in all tissues of *P*. *bracteatum* L., and we could not detect miR1310 in root, stem and capsule tissues of this species. Interestingly, among all of the clustered mature miRNAs, only miR2911 was detected in stem tissue of *P*. *bracteatum* L. and had the highest expression level than other tissues of this species. We also observed the maximum expression level of miR2910, miR2914 and miR2916 in root and leaves tissues of *P*. *bracteatum* L.. The miR1310 had the highest expression level in capsule and leaves tissues of *P*. *somniferum* L., while, miR2911, miR1310 and miR2914 showed the maximum expression level in capsule tissue of *P*. *somniferum* L. In contrast, only miR2914 had the highest expression level in capsule tissue of *P*. *bracteatum* L. The difference expression of this clustered mature miRNAs was statistically significant in each tissue of two species (*P* < 0.001, Fisher's exact test).

The results illustrate the complicated patterns of mature and precursor miRNAs expression. There was no certain relationship between the expression of mature miRNAs and their precursors. Additionally, the expression pattern of mature miRNA would be different than precursor miRNA, as far as concrete evidences are concerned [45–49].

### Target gene prediction and GO functional analysis

Computational approaches of miRNA target predictions can provide insights in elucidation miRNA functions. In this study, most of the miRNA target genes are involved in a range of diverse cellular, physiological, developmental and metabolic pathways, in addition to signal sensing and phytohormone signal cascades, organic acid and glutamate biosynthesis, cell wall synthesis and biotic/abiotic stresses (see S1 File). Interestingly, 21 miRNAs are predicted to target 284 genes that are in various important biological processes (see S1 File). The KEGG analysis suggested that predicted miRNAs were involved in various metabolic pathways such as phosphatidylinositol signaling, lipopolysaccharide, phenylpropanoid, glycosphingolipid and steroid hormone biosynthesis (S2 Table).

According to target prediction, the protein target of some of the miRNAs including miR2910, miR2911, miR2914, miR2916, miR415 and miR414 are associated with abiotic stress responses. These protein targets are related to cell-to-cell mobile that has been involved in embryo development ending in seed dormancy, plasmodesma organization and RNA secondary structure unwinding. Additionally, transcription factors (TFs) were the major miRNA targets. In this regard, MYB, zinc finger and Homeobox-leucine zipper protein (HD-ZIP) TF families were predicted as targets of pso-miR2914, pso-miR415, pso-miR414 and pso-miR2910, pso-miR414, pso-miRf10082-akr, and pso-miR172b miRf10082-akr (Fig 3). TFs are known as an important cellular proteins, which play pivotal a role in a number of biological processes. It has been proved that TFs modulate biotic and abiotic stress responses by activating many genes that play important and effective roles in controlling of a diverse biological processes [50–56]. Our results correspond to an earlier report in maize and strawberry [57, 58]. Transcript of F-box protein which is targeted by miR2911 was annotated to be involved in the response to cold and heat stresses in *P*. *somniferum* L. (see S1 File). Moreover, gene targets of miR1310 also including RNA helicase, chaperonin CPN60-1 and protein kinase were predicted (Table 3). In this study, high-level expression of pso-miR2914 was observed in the stem of *P*. *somniferum* L. that it might be because of its role in primary and secondary metabolite control. It is noteworthy that this tissue contains different alkaloids metabolite pathways such as noscapine. In other word, production and accumulation of noscapine occurred in stem of opium poppy rather than that is in other tissues [59] which might be regulated by different factors such as TFs and miRNAs [60]. The miR2914 was predicted to target R2R3 MYB TFs. MYB proteins, among the largest TF superfamilies in eukaryotes [61], function in diverse biological processes including regulation of primary/secondary metabolisms, control of cell cycle and development, biotic/abiotic stress responses, hormone synthesis, and signal transduction [62–64] (Table 3).

**Table 3:** Some of the putative targets for clustered miRNAs in *P*. *somniferum* L.

miRNA family	Target ID	Annotation	Contig ID
miR1310	AT1G12770	nucleoside triphosphate hydrolases protein	Contig_2022
	TC274430	Squalene monooxygenase	Contig_2000
	TC275327	Chaperonin CPN60-1, mitochondrial	Contig_2001
	TC549092	Metallothionein-like protein 1A	Contig_2002
	TC555807	Ribulose-phosphate 3-epimerase	Contig_2003
miR2910	AT5G18700	Protein kinase	Contig_2023
	AL506563	Glucose-1-phosphate adenylyltransferase	Contig_2004
	Medtr4g082260.1	Anaphase-promoting complex subunit	Contig_2005
	TC11669	ATPase, H+ transporting, V1 subunit B2	Contig_2006
	TC20580	xyloglucan endotransglucosylase/hydrolase 1	Contig_2007
	TC21381	Tyrosine decarboxylase	Contig_2008
	TC124689	ADH-like UDP-glucose dehydrogenase	Contig_2009
	TC442409	DEAD-box ATP-dependent RNA helicase	Contig_2015
miR2914	AT2G19940	Oxidoreductases	Contig_2024
	AT1G74540	CYP98A8	Contig_2025
	LOC_Os05g49760.1	Dehydrogenase	Contig_2010
	TC189252	Phosphoglycerate kinase	Contig_2011
	TC232414	Fructose-bisphosphate aldolase	Contig_2012
	TC395503	N-acetyl-gamma-glutamyl-phosphate reductase	Contig_2013
	TC505425	Typical P-type R2R3 Myb protein	Contig_2014
miR2916	Medtr2g018660.1	Hydrolase-like protein	Contig_2016
	TC20258	2,3-bisphosphoglycerate mutase	Contig_2017
	TC48496	5-methyltetrahydropteroyltriglutamate-homocysteine methyltransferase	Contig_2018
	TC49012	Methionine synthase	Contig_2019
miR2911	AT1G21760	Cyclin-like F-box domain containing protein	Contig_2026
	TC4115	ADP-ribosylation factor	Contig_2020
	TC209201	Cytochrome P450	Contig_2021

Previous studies showed that many of cytochrome and oxidoreductase enzymes act as abiotic-responsive functional genes in plant cells [65, 66]. For instance, an *oxidoreductases* gene which involves in phosphate-reductase, ion and NADP or NAD binding activity, was predicted to be the target of miR2914 (S5A Fig). Another predicted target gene of miR2914 is *CYP98A8*, which functions in phenylpropanoid biosynthesis pathway (S5B Fig).

**Fig 6:**
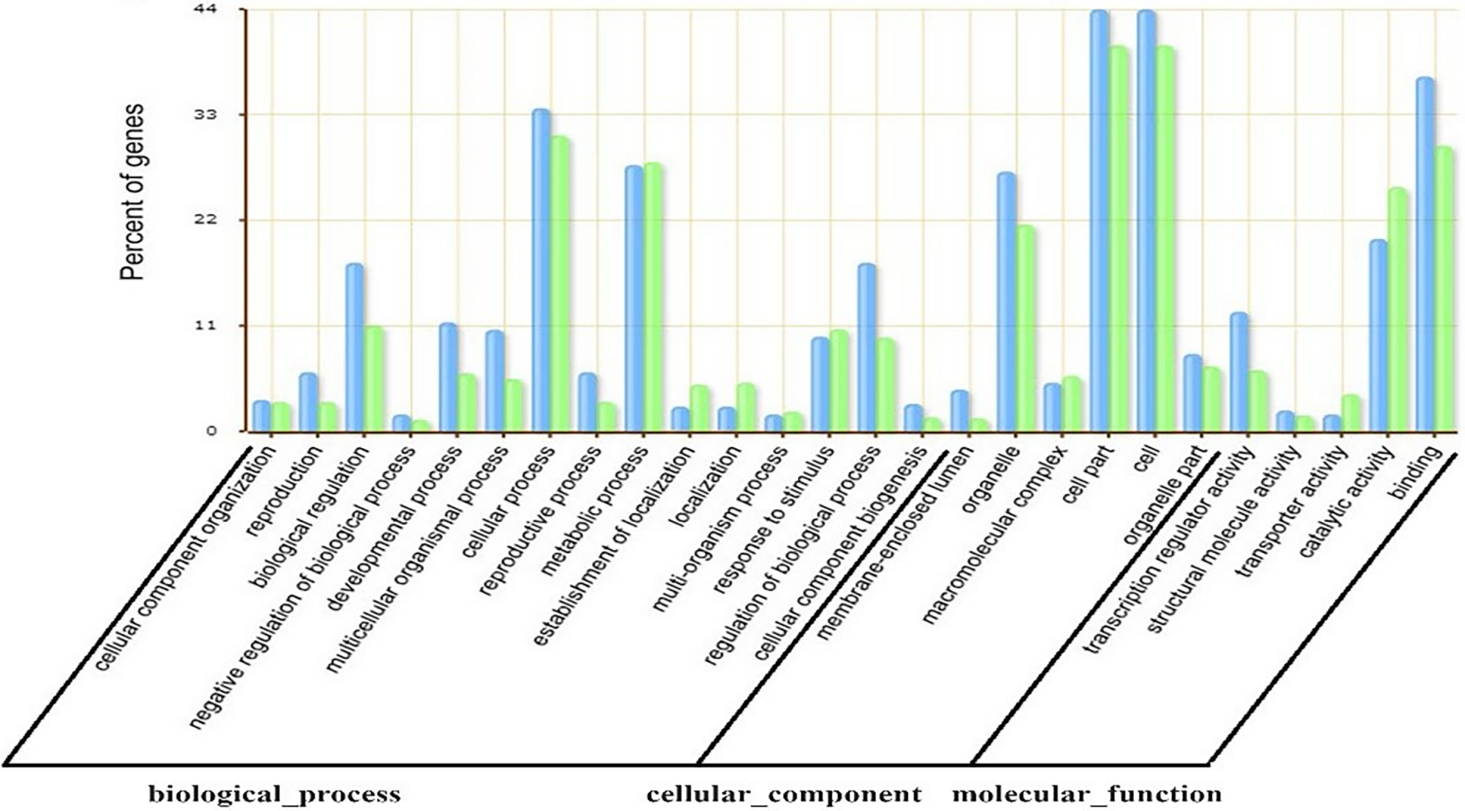
Gene categories and distribution of miRNA targets in *P*. *somniferum* L.

Herein, we also constructed a simple network for two genes targeted by miR2914. According to co-expression network analysis, *CYP98A8* gene is co-expressed with genes involved in other biological processes such as monoterpene, flavonol and spermidine biosynthesis pathways, and *oxidoreductases* gene is also co-expressed with oxidative-responsive factors in plants and genes involved in arginine biosynthesis, response to cadmium ion, oxidation-reduction process and cellular amino acid metabolisms (S5A Fig). Subsequently, GO enrichment analysis using TAIR and agriGO databases was performed to reduce complexity and highlight biological processes associated with the miRNAs targets. (Fig 6) summarized the categorization of miRNA-target genes according to the cellular component, molecular function, and biological process. The GO molecular function demonstrated that miRNA targets may be involved in diverse molecular functions including binding (GO:0005488), catalytic activity (GO:0003824), transcription regulatory activity (GO:0030528), structural molecule activity (GO:0005198) and transport activity (GO:0005215). Among the GO terms, the predominant terms of the cellular component are involved in cell part (GO:0044464), cell (GO:0005623, organelle (GO:0043226). For biological processes, those assignments were mostly given to cellular processes (GO:0009987) 31%, metabolic processes (GO:0008152) 26%, biological regulation (GO:0065007) 17% and developmental processes (GO:003502) 11% percent of genes (Fig 6).

Subsequently, to identify the target genes of clustered miRNAs in the *P*. *somniferum* L. transcriptome, we used several RNAseq projects (http://www.ncbi.nlm.nih.gov/sra/ *Papaver somniferum* L.). RNA-seq reads that had high similarity to the previously known predicted target genes were selected using the Offline BLAST software and were subsequently used to build up consensus sequences utilizing the Codon Code Aligner v. 5.0.1. program. Next, the target region of miRNAs of each chosen consensus sequence was found. 27 putative miRNA target genes of clustered miRNAs were listed in S1 Table.

## Discussion

MiRNAs play important roles in plant key biological processes such as growth, development, signal transduction and environmental stress responses [67]. More recently, some miRNAs related to gene regulation of secondary metabolites, reported in *P*. *somniferum* L. [22, 23]. The development of miRNA identification and expression analysis, including direct cloning, EST analysis and deep sequencing techniques, has provided opportunities for studying of miRNAs.

The RNA-seq reads analysis in Papaveraceae led to the identification of a clustered miRNA located in a long polycistronic pre-ribosomal RNA. According to the previous studies, miR2910, miR2911, miR2914 and miR2916 were identified by directional cloning from *P*. *euphratica* Oliv. [44], also miR1310 was isolated from *Pinus contorta* Douglas. by small RNA sequencing [68]. Moreover, identification and expression analysis of the five miRNAs was achieved by mature miRNA sequencing [23]. In this study, we observed high similarity on the BLAST hits, more analysis indicated to conservation of five pre-miRNAs. Following, we could identify the location of the clustered microRNA in the pre-ribosomal RNA. Several investigations indicate that miRNAs are generated from snoRNAs [69], endogenous-siRNAs [70] and tRNA-like structures [71], as well as the examinations of mouse antisense-miRNA have revealed that mmu-miR712 and hsa-miR663 are originated of pre-ribosomal RNA [14].

Precursors of plant miRNAs have larger and more variable stem-loop than animals [72], and the conservation of mature miRNAs in plants is higher than that of precursor sequences [73]. In order to elucidate evolutionary analysis of the clustered miRNAs, we compared clustered miRNAs from 14 species RNA-seq read projects. Interestingly, comparative analysis of orthologous miRNAs among 14 plant species suggest all members of the clustered miRNAs have high homology in both mature and precursor miRNAs. The main reason for this similarity, is that these miRNAs are located in the region of the genome where is highly conserved. It may hint that the miRNA clustering is important for coordinate regulation of gene expression in plant species containing this miRNA cluster [74]. Earlier studies identified a few clustered miRNA genes in *Taxus chinensis* Render. [20], *Panicum virgatum* L. [21] and some other plant species [75]. Due to the high conservation of both mature and precursor clustered miRNAs in various studied species, it could be suggested that there is ancient coevolution between miRNAs and ribosomal RNA as well as providing valuable evidence showing that the clustered miRNAs would play crucial roles in studied species. In other words, transcript of pre-rRNA shows dual molecular functions that could act as a miRNA and rRNA. Indeed, a transcript unit contains five miRNA genes that after cleavage to mature miRNAs, are able to directly repress the expression of hundreds of genes at the post-transcriptional level. Some of *P*. *somniferum* L. miRNAs were also identified in *P*. *bracteatum* L. indicating that the majority of the identified opium poppy miRNAs could be expressed in Persian poppy (Table 3). In addition, we realized that the sequences of five predicted pre-miRNAs including miRf12256, miR1122, miR415, miR6485 and miRf10082 are highly conserved between *P*. *somniferum* L. and *P*. *bracteatum* L. species. Furthermore, target prediction and GO analysis revealed their roles in a variety of developmental and biological processes. The miR2914 (a member of clustered miRNA) triggers *CYP98A8* which is a member of *CYP450s* gene family involved in the phenolamide pathway and formation of major pollen coat compounds [76] and phenolic pathway in the flowers of *A*. *thaliana* L. [77]. Additionally, it has been reported that *CYP71* which is a target of miR5023, is involved in terpenoid biosynthesis [78]. According to the previous investigations, cytochrome P450s gene families are mostly involved in the biosynthesis of hormones, phytoalexins, diverse terpenoid, lignins, fatty acids and sterols [79, 80]. R2R3 MYB transcription factor that is regulated by miR2914, plays important roles in growth, development and plants secondary products’ biosynthesis pathways such as phenylpropanoid biosynthesis [81], isoflavone [82]. In addition to gene silencing mediated by RNA, new technologies like clustered regularly interspaced short palindromic repeats (CRISPR)/CRISPR-associated protein 9 (Cas9) have been used for targeted down-regulating of gene expressions. The 4′OMT2 gene, which involved in biosynthesis of benzylisoquinoline alkaloids (BIAs) in *P*. *somniferum* L. has been edited by CRISPR/Cas9 technology. As a result 4′OMT2 expression has been significantly reduced and subsequently amount of BIAs decreased as well as a new uncharacterized metabolite has been appeared in metabolite content [83].

In the frame of this work, we also measured the transcript level of precursor and mature clustered miRNAs in four tissues in *P*. *somniferum* L. and *P*. *bracteatum* L. using qRT-PCR. Mature miRNA is part of an active RNA-induced transcriptional silencing (RITS) and quantification of the active mature miRNA, rather than the inactive pre-miRNA, is preferred. Therefore, quantification of miRNA precursor was also used to validate the results obtained from mature miRNA profiling experiments [84–86].

Based on obtained results, the particular relationship between the expression of precursor and it’s corresponding mature of studied clustered miRNAs was not observed. As we know, the expression level of pre-ribosomal RNA as a housekeeping gene, which harbored the clustered miRNAs, is very high. Moreover, the most precursors of clustered miRNAs in most tissues of both species were expressed at high level unlike their corresponding mature miRNA expressions that had almost no expression or expression at a very low level in some tissues of both species (Figs 3–5). The reasons for different expression pattern of precursor and mature miRNAs could be related to post-transcriptional regulatory mechanisms, which may function at multiple processing steps in miRNA biogenesis and control miRNA expression, such as processing of precursor miRNA by DCL1 endonuclease which is accompanied by other microprocessor proteins and generating mature miRNA/miRNA* duplexes. It should be mentioned that these processing events carry out in the nucleus of plant cells [87–89]. Previous studies have clarified that miRNA genes that are physically close to each other, are transcribed as clustered units that regulate the expression of genes involved in the control of various biological processes [21, 90]. The question can be raised here is that why these miRNAs are located in pre-rRNA, which is expressed in every cell at all times as well as at very high level? The answer to this question may lie in the requirement of cells for high-level expression of the miRNAs due to their crucial functions in important biological processes. Based on the outcome of GO and computational target prediction analysis, the major of the putative target of clustered miRNAs were predicted to be related to cellular and metabolic processes as well as developmental regulation. The splicing mechanism of pre-rRNA is quite unknown in plants. According to recent studies, mechanisms of miRNA biogenesis are divided into canonical and non-canonical pathways [91, 92]. Non-canonical miRNAs were recognized to have various origins, including introns, snoRNAs, endogenous shRNAs, rRNAs and tRNAs. Divers non-canonical pathways generate the majority of animal miRNAs. Non-canonical miRNA biogenesis pathways are illustrated by evidence from animal and plant miRNAs diversity [93]. Pre-rRNAs are the universal noncoding RNAs in eukaryotes that have to be processed by various endonucleolytic cleavages, which are necessary for a maturation of 18S, 5.8S and 28S rRNA in nucleus and cytoplasm [94]. This study indicated that the clustered miRNAs may be emerged by a non-canonical pathway from pre-rRNA in the plant kingdom. However, further studies are needed for a better understanding of mechanisms causing rRNA maturation and miRNAs production. Discovering these mechanisms will provide an improvement in understanding of gene regulation mediated by clustered miRNAs.

## Supporting information

S1 Fig The secondary structure of miRNAs in *P*. *somniferum* L. and *P*. *bracteatum* L. (DOCX)

S2 Fig The exact location of the clustered miRNAs in *P*. *somniferum* pre-RNA. (TIFF)

S3 Fig DNase I treatment SDR gene. M, 100 bp DNA ladder (SMOBIO: dm2300) is applied as molecular weight marker, separated by electrophoresis.
(TIFF)

S4 Fig Gel blot analyses of clustered miRNAs. Amplification of fragments of miR2910 (PM1), miR2914 (PM2), miR2916 (PM3), miR2911 (PM4), miR1310 (PM5), PCR positive control 5.8S RNA (C+) and negative control with no cDNA template (C-), M, 100 bp DNA ladder (SMOBIO: dm2300) is applied as molecular weight marker, separated by electrophoresis.
(TIFF)

S5 Fig Co-expression, network analysis of targets of miR2914 in Arabidopsis dataset. (A) The hypothetic network of (A) *Oxidoreductase* gene, (B) *CYP98A8* genes (B).
(TIFF)

S1 Table Lists of the potential targets genes for clustered miRNAs in *P*. *somniferum* L and *P*. *bracteatum* L. (DOCX)

S2 Table KEGG analysis of miRNA targets in *P*. *somniferum* L. and *P*. *bracteatum* L. (DOCX)

S1 File The sequence of pre-miRNA. (XLSX)
