# Supplementary figures and images for: Identification and expression analysis of a microRNA cluster derived from pre-ribosomal RNA in Papaver somniferum L. and Papaver bracteatum L
Source: PLoS One. 2018 Aug 1;13(8):e0199673. doi: 10.1371/journal.pone.0199673 (PMC6070170; doi:10.1371/journal.pone.0199673)

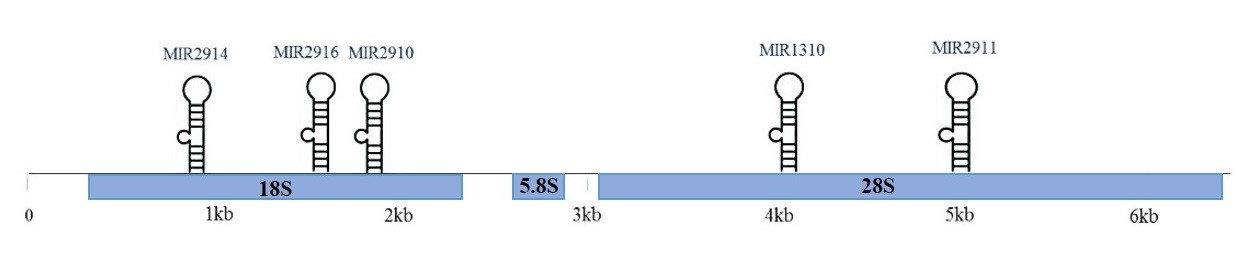

Supplement: S2 Fig — (TIFF) [file pone.0199673.s002.tiff]

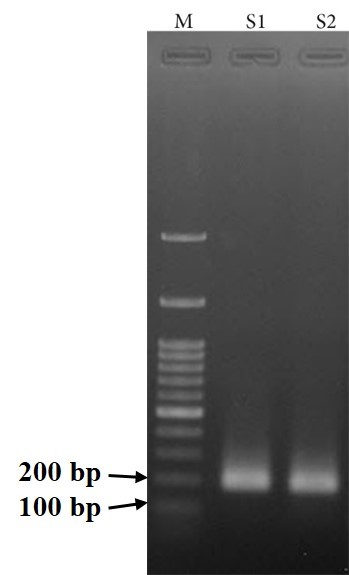

Supplement: S3 Fig — M, 100 bp DNA ladder (SMOBIO: dm2300) is applied as molecular weight marker, separated by electrophoresis. (TIFF) [file pone.0199673.s003.tiff]

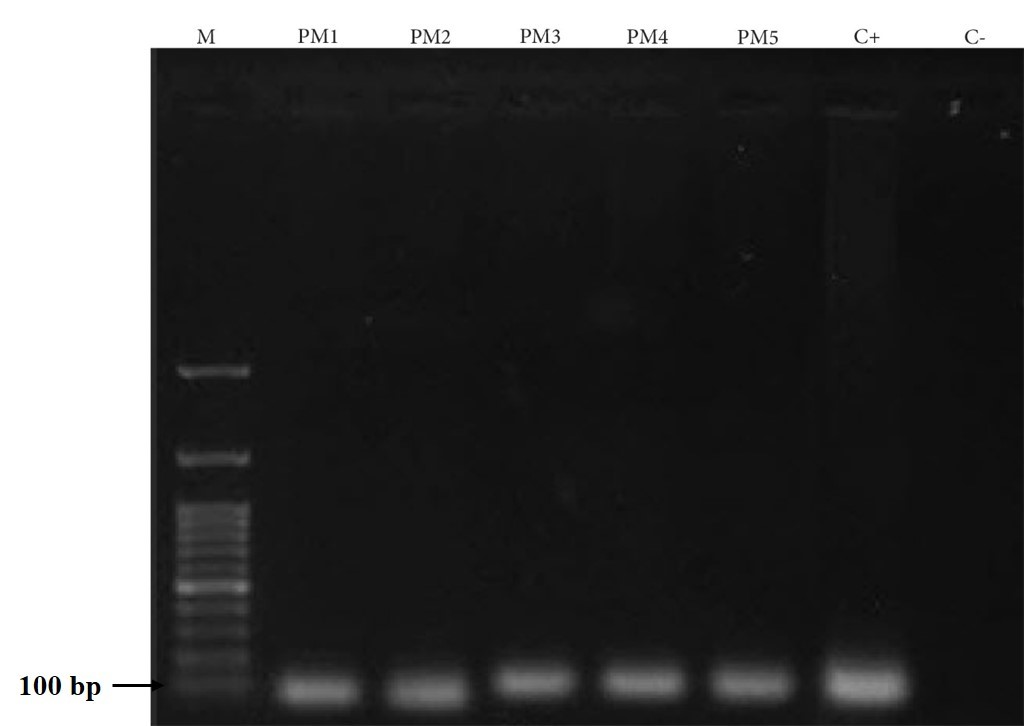

Supplement: S4 Fig — Amplification of fragments of miR2910 (PM1), miR2914 (PM2), miR2916 (PM3), miR2911 (PM4), miR1310 (PM5), PCR positive control 5.8S RNA (C+) and negative control with no cDNA template (C-), M, 100 bp DNA ladder (SMOBIO: dm2300) is applied as molecular weight marker, separated by electrophoresis. (TIFF) [file pone.0199673.s004.tiff]

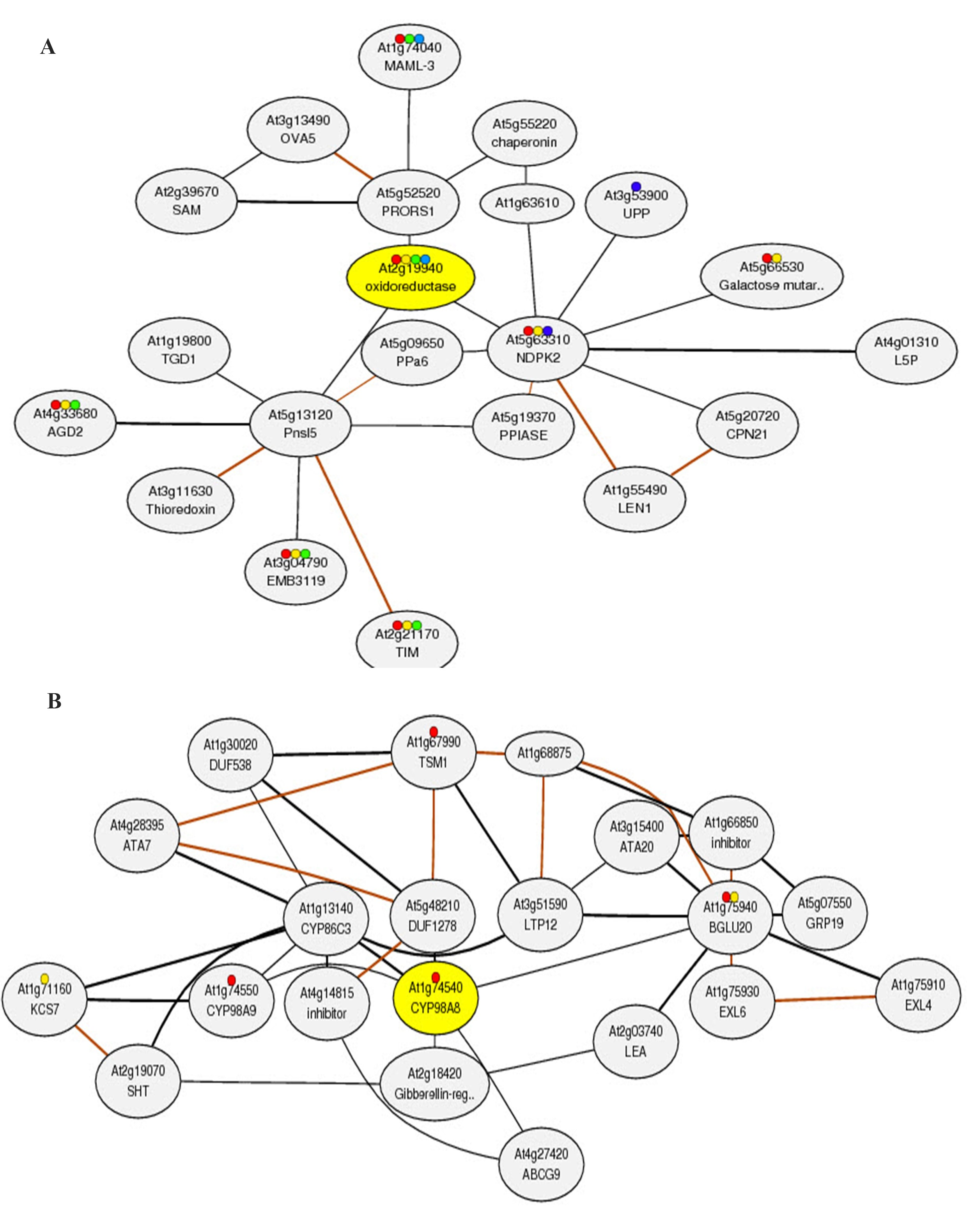

Supplement: S5 Fig — (A) The hypothetic network of (A) Oxidoreductase gene, (B) CYP98A8 genes (B). (TIFF) [file pone.0199673.s005.tiff]
