# Supplementary material for: Identification and expression analysis of a microRNA cluster derived from pre-ribosomal RNA in Papaver somniferum L. and Papaver bracteatum L
Source: PLoS One. 2018 Aug 1;13(8):e0199673. doi: 10.1371/journal.pone.0199673 (PMC6070170; doi:10.1371/journal.pone.0199673)
Supplement: S1 Table — (DOCX) [file pone.0199673.s006.docx]

| miRNA family | Target ID |  |
| --- | --- | --- |
| pso-miR1310 | AT1G12770 | miRNA 23 UCCCCGCAA-UGCGGGGGCUACGG 1  :: ::::: ::::::.:::::::  Target 1469 AGAUGCGUUCACGCCCUCGAUGCC 1492 |
|  | TC274430 | miRNA 21 CCCGCAAUGCGGGGGCUACGG 1  :::::::.:::::::::::::  Target 282 GGGCGUUGCGCCCCCGAUGCC 302 |
|  | TC275327 | miRNA 21 CCCGCAAUGCGGGGGCUACGG 1  :::::::.:::::::::::::  Target 1626 GGGCGUUGCGCCCCCGAUGCC 1646 |
|  | TC549092 | miRNA 21 CCCGCAAUGCGGGGGCUACGG 1  :::::::.:::::::::::::  Target 392 GGGCGUUGCGCCCCCGAUGCC 412 |
|  | TC555807 | miRNA 21 CCCGCAAUGCGGGGGCUACGG 1  :::::::.:::::::::::::  Target 338 GGGCGUUGCGCCCCCGAUGCC 358 |
| pso-miR2910 | AT5G18700 | miRNA 20 UGUUUAGCGAGGUGGUUGAU 1  ::::::.:::.:::.::.:.  Target 1854 ACAAAUUGCUUCACUAAUUG 1873 |
|  | AL506563 | miRNA 20 UGUUUAGCGAGGUGGUUGAU 1  ::: ::: ::.::::::.::  Target 546 ACACAUCCCUUCACCAAUUA 565 |
|  | Medtr4g082260.1 | miRNA 21 CUGUUUAGCGAGGUGGUUGAU 1  :::::::: ::::: ::::::  Target 538 GACAAAUCACUCCAACAACUA 558 |
|  | TC11669 | miRNA 21 CUGUUUAGCGAGGUGGUUGAU 1  :::::::. ::::::::.::  Target 550 GACAAAUUUAUCCACCAAUUA 570 |
|  | TC20580 | miRNA 21 CUGUUUAGCGAGGUGGUUGAU 1  :::::::::::::::::::::  Target 387 GACAAAUCGCUCCACCAACUA 407 |
|  | TC21381 | miRNA 20 UGUUUAGCGAGGUGGUUGAU 1  :::.::. ::::::::::::  Target 747 ACAGAUUCCUCCACCAACUA 766 |
|  | TC124689 | miRNA 21 CUGUUUAGCGAGGUGGUUGAU 1  ::: :::.::: .::::::::  Target 1247 GACUAAUUGCUAUACCAACUA 1267 |
|  | TC442409 | miRNA 21 CUGUUUAGCGAGGUGGUUGAU 1  :::::::::::::::::::::  Target 389 GACAAAUCGCUCCACCAACUA 409 |
| pso-miR2914 | AT2G19940 | miRNA 20 GCAGUGGGCAGUGGUGGUAC 1  .:::::::::::.:.:::::  Target 914 UGUCACCCGUCAUCGCCAUG 933 |
|  | AT1G74540 | miRNA 20 GCAGUGGGCAGUGGUGGUAC 1  : :::::::::: ::::::  Target 1110 CUUCACCCGUCAACACCAUU 1129 |
|  | LOC_Os05g49760.1 | miRNA 21 GGCAGUGGGCAGUGGUGGUAC 1  :.:::::::::::..:::.::  Target 1041 CUGUCACCCGUCAUUACCGUG 1061 |
|  | TC189252 | miRNA 23 GAGGCAGUGGGCAGUGGUGGUAC 1  :::::::::::::::::::::::  Target 386 CUCCGUCACCCGUCACCACCAUG 408 |
|  | TC232414 | miRNA 23 GAGGCAGUGGGCAGUGGUGGUAC 1  ::::::::::::::::::::::  Target 1040 CUCCGUCACCCGUCACCACCAUA 1062 |
|  | TC395503 | miRNA 20 GCAGUGGGCAGUGGUGGUAC 1  .:::::::::::.:.:::::  Target 913 UGUCACCCGUCAUCGCCAUG 932 |
|  | TC505425 | miRNA 23 GAGGCAGUGGGCAGUGGUGGUAC 1  :::::::::::::::::::::::  Target 1149 CUCCGUCACCCGUCACCACCAUG 1171 |
| pso-miR2916 | Medtr2g018660.1 | miRNA 21 UACUAGCAGAAGCUCAGGGGU 1  ::::: ::.: ::::::::::  Target 932 AUGAUGGUUUGCGAGUCCCCA 952 |
|  | TC20258 | miRNA 23 UAUACUAGCAGAAGCUCAGGGGU 1  :: ::::::: : ::::::::.:  Target 175 AUUUGAUCGUGUACGAGUCCCUA 197 |
|  | TC48496 | miRNA 21 UACUAGCAGAAGCUCAGGGGU 1  :::: ::::::: .::::.::  Target 220 AUGACCGUCUUCUGGUCCUCA 240 |
|  | TC49012 | miRNA 21 UACUAGCAGAAGCUCAGGGGU 1  :::: ::::::: .::::.::  Target 1264 AUGACCGUCUUCUGGUCCUCA 1284 |
| pso-miR2911 | TC4115 | miRNA 20 AGGGUCGGGCAGGGGGCCGG 1  ::::. :::::.:.:::::.  Target 38 UCCCGUCCCGUUCUCCGGCU 57 |
|  | TC209201 | miRNA 20 AGGGUCGGGCAGGGGGCCGG 1  ::::::.:::::::::::::  Target 369 UCCCAGUCCGUCCCCCGGCC 388 |
