# Supplementary material for: Identification and expression analysis of a microRNA cluster derived from pre-ribosomal RNA in Papaver somniferum L. and Papaver bracteatum L
Source: PLoS One. 2018 Aug 1;13(8):e0199673. doi: 10.1371/journal.pone.0199673 (PMC6070170; doi:10.1371/journal.pone.0199673)
Supplement: S2 Table — (DOCX) [file pone.0199673.s007.docx]

| **Pathways** | **Accession ID for targets** | **Targets** | **miRNA** |
| --- | --- | --- | --- |
| Pentose and glucuronate interconversions  Starch and sucrose metabolism | AT4G23820.1 | polygalacturonase | MIR6485 |
| Phosphatidylinositol signaling system  Inositol phosphate metabolism | AT3G55940.1 | phosphoinositide phospholipase c 7 | MIR1122 |
| Porphyrin and chlorophyll metabolism | AT2G26550.1 | heme oxygenase | MIR414 |
| Purine metabolism  Thiamine metabolism | AT5G55300.2 | DNA topoisomerase 1 | MIR414 |
| Lipopolysaccharide biosynthesis | AT1G79500.5 | 2-3-dehydro-deoxyphosphooctonate aldolase 1 | MIR414 |
| Porphyrin and chlorophyll metabolism  Starch and sucrose metabolism  Metabolism of xenobiotics and Retinol  Ascorbate and aldarate metabolism  Pentose and glucuronate interconversions  Steroid hormone biosynthesis | AT4G33330.1 | udp-glucuronate:xylan alpha-glucuronosyltransferase 2-like i | MIR414 |
| Purine metabolism | AT5G47040.1 | protease | MIR414 |
| Glutathione metabolism  Phenylpropanoid biosynthesis | AT1G03780.3 | protein tpx2 | MIRf10082-akr |
| Glycosphingolipid biosynthesis - globo series -lacto and neolacto series | AT2G15360.1 | fucosyltransferase 4 | MIR2914 |
| Glycosphingolipid biosynthesis - lacto and neolacto series | AT2G19940.2 | Oxidoreductases | MIR2914 |
| Pentose and glucuronate interconversions | AT3G07010.1 | Pectin lyase-like superfamily protein | MIR2914 |
