# Supplementary material for: Identification and expression analysis of a microRNA cluster derived from pre-ribosomal RNA in Papaver somniferum L. and Papaver bracteatum L
Source: PLoS One. 2018 Aug 1;13(8):e0199673. doi: 10.1371/journal.pone.0199673 (PMC6070170; doi:10.1371/journal.pone.0199673)
Supplement: Supplementary file 4 [file pone.0199673.s001.docx]

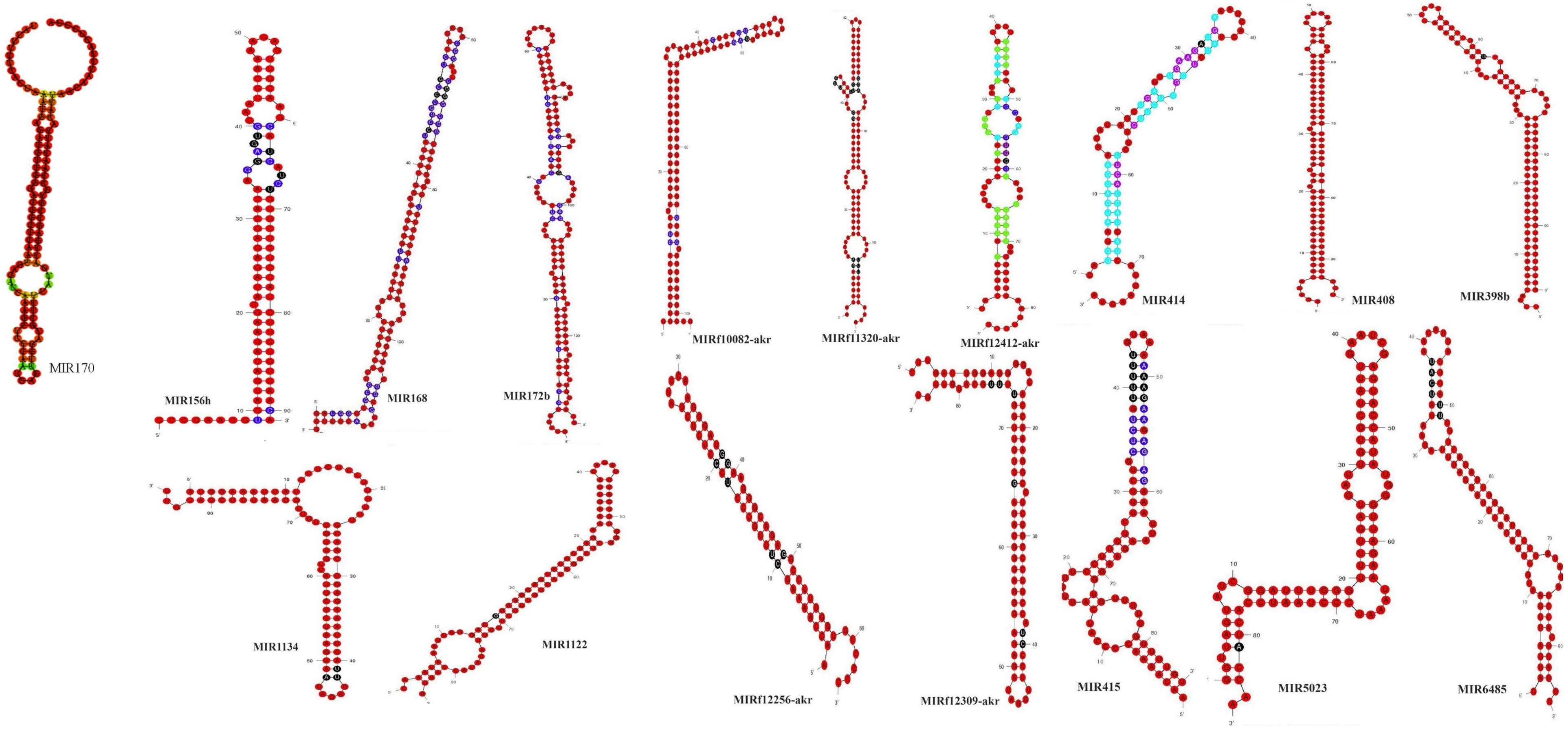
MIR170
MIR156h
MIR168
MIR172b
MIRf10082-akr
MIRf11320-akr
MIRf12412-akr
MIR414
MIR408
MIR398b
MIR1134
MIR1122
MIRf12256-akr
MIRf12309-akr
MIR415
MIR5023
MIR6485
